# Prevalence of and factors associated with female child marriage in Indonesia

**DOI:** 10.1371/journal.pone.0305821

**Published:** 2024-07-05

**Authors:** Heri Kuswanto, Pratnya Paramitha Oktaviana, Ferry Efendi, Nelwati Nelwati, Hema Malini

**Affiliations:** 1 Department of Statistics, Institut Teknologi Sepuluh Nopember, Surabaya, Indonesia; 2 Department of Actuarial Science, Institut Teknologi Sepuluh Nopember, Surabaya, Indonesia; 3 Faculty of Nursing, Universitas Airlangga, Surabaya, Indonesia; 4 Faculty of Nursing, Andalas University, Padang, Indonesia; University of Environment and Sustainable Development, GHANA

## Abstract

Statistics from the 2018 National Social and Economic Survey revealed that one out of nine young females in Indonesia have been in female child marriage, and the prevalence remains high. Considering the serious consequences of female child marriage and that Sustainable Development Goal 5 on gender equality has targeted the elimination of female child marriage by 2030, a study concerning the prevalence and determinants of female child marriage needs to be conducted in Indonesia. In this paper, we examined the prevalence of and factors associated with female child marriage in Indonesia using binary logistic regression. We examined data from the Indonesia Demographic and Health Survey conducted in 2017. A sample of 9,333 young females aged 15–20 years was included in the study. Our analysis involved descriptive and binary logistic regression analysis. The results are presented in percentages and odds ratios (OR), with their respective confidence intervals. Our findings indicate that health insurance and sex of household head did not significantly influence female child marriage. The prevalence of female child marriage in Indonesia was quite high, reaching about 12.53%. Females with no education [OR = 76.448; (CI = 29.73–196.70)], not working [OR = 1.662; (CI = 1.41–1.94)], those with the poorest wealth index [OR = 3.215; (CI = 2.336–4.425)], those living in the east of Indonesia [OR = 1.451; (CI = 1.132–1.862)], and those living in rural areas [OR = 0.718; (CI = 0.609–0.844)] had the higher odds of experiencing female child marriage. Meanwhile, females with a secondary education level [OR = 16.296; (CI = 11.098–23.930)], those with a rich wealth index [OR = 1.940; (CI = 1.404–2.681)], and those living in the middle of Indonesia [OR = 1.263; (CI = 1.074–1.487)] were less likely to experience female child marriage. Educational background was the most significant factor influencing the high prevalence of female child marriage in Indonesia. Female empowerment through education as well as poverty alleviation were factors that could be strengthened to ensure that female child marriage is reduced or eliminated in Indonesia. Equality of access to information and better quality of education also need to be prioritized.

## Introduction

Indonesia is one of the most populated countries in the world, where the demographic pattern is dominated by a young population age [1]. This demography will be a beneficial asset if it can be managed well. Many years ago, the government developed a family planning program to control the population growth rate called Keluarga Berencana (KB), which means that every family (especially newly married couples) were encouraged to have a maximum of two children [2]. However, the program did not work well in some regions due to various factors such as cultural reasons, family background, etc. In line with this program, the government committed to reducing the number of child marriages, especially for females.

According to the United Nations International Children’s Emergency Fund (UNICEF) in 2009, female child marriage (FCM) was defined as the marriage of a female below the age of 18 years. Under Indonesia’s 1974 Marriage Law, marriage is allowed only for males of 19 years old and females reaching 16 years old. However, since September 2019, the legislative revised the bottom limit of ages for marriage of males and females to 19 years old. In fact, many couples marry before 19 years of age. The applications for underage marriage received by the government reached about 34,000 within the first six months of 2020. Of those numbers, 97% of proposals were approved and 60% of them were below 18 years old. Moreover, Indonesia has been listed as having the seventh highest number of child marriages in the world [3], and hence, FCM is a serious issue.

FCM is an important issue due to its prevalence and consequences. More importantly, Forte et al. [4] and Bolarinwa et al. [5] pointed out that FCM violates young women’s fundamental human rights, and it is one of the targets of the Sustainable Development Goals (SDGs), i.e., the elimination of child marriage by 2030. Therefore, to achieve this SDG target, the prevalence of FCM must be reduced by 10% every year from roughly 23% [4]. Furthermore, FCM is also a main factor in increased maternal mortality among adolescent girls [6]. Gibbs et al. [7] found strong evidence that FCM exposes girls to negative health outcomes like maternal morbidity, pregnancy complications, and birthing difficulties.

Several studies have found that FCM may induce additional negative effects. Brown [8] explained how child marriage can lead to a life of poverty, illiteracy due to school dropout and powerlessness for girls in Africa. Furthermore, Kabir et al. [9] conducted a study in Bangladesh and found that early marriage leads to poor sexual and reproductive health. Early marriage has enormous harmful effect on women’s health as they are not ready physically and psychologically, which thus increases the risk for different sexually transmitted diseases, and other maternal related problems such as obstetric fistulas, pre-term deliveries as well as miscarriage accompanied by mental depression. Meanwhile, comprehensive studies have been conducted by [10,11] which pointed out that younger maternal age increases the risk of having low birth weight and increasing chance of premature birth, as well as subpar child nutrition and health outcomes. The findings were based on five birth cohort studies involving five different countries e.g. Brazil, Guatemala, India, the Philippines, and South Africa. A study by de Groot et al. [12] demonstrated that FCM exacerbates girls’ vulnerability to all forms of violence (physical, emotional, and sexual). Using data from Nigeria, Braimah [13] found that FCM causes a high prevalence of vesicovaginal fistula due to all types of violence (physical, emotional, and sexual). Kidman [14] pointed out that married girls are more vulnerable to domestic violence.

Using data from the Indonesia Family Life Survey (IFLS5), Cameron et al. [15] found that Indonesian women who marry before the age of 19 years can anticipate a lesser quality of life for themselves and their children than women who wait a few years. Moreover, three out of ten Indonesian women were reported to have married before they turned 19. Using data covering the period 2010–2015, the Indonesian Statistics Agency (BPS) and UNICEF conducted another study and found that pressing poverty, lack of education and to a certain extent, cultural norms were some of the factors contributing to the prevalence of child marriage in Indonesia [16]. The study found interesting results where girls who marry before age 18 were at least four times less likely to complete secondary education or its equivalent. Furthermore, child marriage is estimated to cost economies at least 1.7% of Gross Domestic Product (GDP), projected based on the loss of cash flow that could have been generated if married girls (aged 15–19 years) delayed marriage until age 20, over the 2014–2050 period. Marshan et al. [17] calculated the prevalence of child marriage using the National Social and Economic Survey (SUSENAS) data. The determining factor for child marriage in women aged 20–24 who were still living with their original household as youngsters was investigated using logistic regression. They discovered several factors, where social and economic features both within and surrounding the girls were the main determinants having a good or negative impact on the likelihood of child marriage incidence. The study therefore suggested that policy should concentrate on how to foster an atmosphere that promotes the development of alternatives to child marriage.

This study aims to investigate the prevalence of and factors associated with FCM in Indonesia using the 2017 Indonesia Demographic and Health Survey (IDHS) data. It differs from Marshan et al. [17] in the sense that we used a more recent dataset from a different source. Moreover, the predictor variables used in the analysis were also different. The response variable we used was current marital status that had two categorical levels and hence, we applied binary logistic regression.

## Materials and method

### Data source, sample size, and inclusion criteria

The data analyzed in this research were from the 2017 IDHS. The dataset is available online at https://doi.org/10.7910/DVN/PWKVMX. The dataset comprised 49,627 respondents in total. However, for the analysis, the data were filtered by including only females aged 15–20 years old, resulting in 9,333 respondents. This research project received ethical clearance and approval from the Research and Ethics Committee of the Institut Teknologi Sepuluh Nopember, Indonesia.

### Study variables

The response (outcome) variable was current marital status (at the time of survey) with two categories, i.e., never in union or even married/living together. The predictor (explanatory) variables were education level, health insurance, occupation, wealth index, province, type of residence, and sex of the household head. These variables were determined through a preliminary study by referring to several published studies as well as considering the availability of the variables of interest in the IDHS datasets. The detailed categories of each variable are summarized in Table 1.

**Table 1:** Variables and its category.

Variable	Definition	Category
Y (current marital status)	The marital status at the time of survey.	0 = never in union 1 = ever married/living together
X_1_ (highest education level)	It is the highest education level of the respondent (the woman surveyed) at the time of survey	0 = no education 1 = primary 2 = secondary 3 = higher
X_2_ (respondent’s occupation)	The respondent’s occupation at the time of survey	0 = not working 1 = working
X_3_ (wealth index)	Households receive scores determined by the quantity and types of consumer goods they possess, including items like televisions, bicycles, or cars, as well as housing attributes such as the source of drinking water, toilet facilities, and flooring materials. Principal component analysis is employed to calculate these scores. National wealth categories are established by assigning the household score to each regular (de jure) member, arranging individuals in the household population based on their scores, and subsequently dividing the distribution into five equal segments, each encompassing 20% of the population.	0 = poorest 1 = poorer 2 = middle 3 = richer 4 = richest
X_4_ (province)	Part of province where the respondent live	0 = East of Indonesia 1 = Middle of Indonesia 2 = West of Indonesia
X_5_ (type of residence)	Urban consists of every city or municipality having a population density of at least 1,000 persons per square kilometer; and Rural is otherwise.	0 = urban 1 = rural
X_6_ (health insurance)	Whether the respondent has health insurance or not	0 = no 1 = yes
X_7_ (sex of household head)	Household head refers to the person who generally provides the chief source of income for the household unit.	0 = male 1 = female

### Data analytical method

#### Logistic regression

Logistic regression is a powerful method to measure the relationship between a categorical response variable with one or multiple explanatory variables. Binary logistic regression is typically used when the dependent variable is dichotomous, and the independent variables are either continuous or categorical. When the dependent variable is not dichotomous and is comprised of more than two categories, a multinomial logistic regression can be employed. The relationship between one explanatory variable *x* and the categorical response variable *y* is expressed in the following form:

$$p(x)=\frac{1}{1+e^{-\alpha -\beta x}}$$ (1)

Eq (1) is known as the function of logistic response, where *α* and *β* are estimated parameters (*β* > 0) and *p*(*x*) is the probability of response *y* (0 ≤ *p*(*x*) ≤ 1). The logistic model is popular because the logistic function, on which the logistic regression model is based, provides estimates in the range 0–1 and an appealing S-shaped description of the combined effect of several risk factors on the risk for an event [18].

#### Odds and odds ratio (OR)

Odds are the ratio of the probability that an event will occur to the probability that it will not occur. If *p* is the probability that an event will occur, then (1*-p*) is the probability that the event will not occur. Then, the corresponding odds is a value given by:

$$odds=\frac{p}{1-p}$$ (2)

Since logistic regression accounts for the difference between the probability that an event will occur and the probability that it will not occur, the effect of independent variables is typically explained in terms of probability. In logistic regression, the average of the response variables *p* in relation to an explanatory variable *x* is modeled in terms of *p* and *x* by the equation *p* = *α* + *βx*. However, this is a poor model because extreme *x* values will produce values of *α* + *βx* that do not lie between 0–1. Logistic regression solves this problem by transforming the odds by the natural logarithm [19]. In logistic regression, the log odds are modeled as a logarithm of the linear function of an explanatory variable:

$$logit(y)=\ln\left(odds\right)=ln\left(\frac{p}{1-p}\right)=\alpha +\beta x$$ (3)

where *p* represents the probabilistic outcome and *x* represents the explanatory variable; and the logistic regression parameters are *α* and *β*.

Eq (3) can be applied to both sides to form an antilog, which can be used to predict the likelihood of an interested outcome:

$$p=p(Y|X=x)=\frac{e^{\alpha +\beta x}}{1+e^{\alpha +\beta x}}=\frac{1}{1+e^{-(\alpha +\beta x)}}$$ (4)

A complex logistic regression can be constructed by extending the logic of the basic logistic regression to include multiple predictors as:

$$logit(y)=ln\left(\frac{p}{1-p}\right)=\alpha +\beta_{1}x_{1}+\cdots +\beta_{k}x_{k}$$ (5)

Therefore,

$$p=p(Y|X_{1}=x_{1},\ldots ,X_{k}=x_{k})=\frac{e^{\alpha +\beta_{1}x_{1}+\cdots +\beta_{k}x_{k}}}{1+e^{\alpha +\beta_{1}x_{1}+\cdots +\beta_{k}x_{k}}}=\frac{1}{1+e^{-(\alpha +\beta_{1}x_{1}+\cdots +\beta_{k}x_{k})}}$$ (6)

The significance of the regression coefficients can be tested by using the Wald statistic, which is defined as:

$$W=\frac{\hat{\beta }}{SE(\hat{\beta )}}$$

where $\hat{\beta }$ is the coefficient estimate and $SE(\hat{\beta )}$ is the standard error estimate of $\hat{\beta }$.

The odds ratio (OR) is a measure of the probability of two events occurring in relation to one another. For two occurrences, A and B respectively, the odds of occurrence of A relative to occurrence of B are:

$$OR[A\;vs\;B]=\frac{odds[A]}{odds[B]}=\frac{P_{A}/(1-P_{A})}{P_{B}/(1-P_{B})}$$ (7)

An OR is a measurement of the association of an exposure with a specific outcome. The OR is the probability that a specific outcome is likely to occur in the presence of a specific exposure compared to the probability that the exposure would not result in the same outcome.

When calculating a logistic regression, the regression coefficient (β) is the estimated increase in the logistic likelihood per unit increase in the independent variable value. The exponential function (*e*^*β*^) of the regression is the increase in the OR of the independent variable per unit increase. The OR can be used to determine whether a particular exposure is associated with a particular outcome, and to compare the size of various risk factors associated with that outcome. OR = 1 indicates that the exposure does not affect the likelihood of the outcome, while OR > 1 indicates exposure associated with a higher likelihood of the outcome. The confidence interval of the odds ratio can be calculated as CI = exp(β^±zα2SE(β^)), where zα2 is a standard normal distribution value with significant level α.

## Steps of the analysis

The outputs presented in this paper are performed by using SPSS software. The steps of the analysis can be described as follows:

- Performing the descriptive statistics of the respondent profile through the percentage of categories in each investigated variable
- Construct the binary logistic model between the response variable (marital status) and the predictors (x1 to x7 as listed in Table 1). In this case, we set the last category as the reference category.
- Estimate the regression coefficients and test the significance of the parameters. We used level of significant (alpha) equals to 5% to decide whether the predictor variable significantly influenced the response or not.
- Calculate the OR and its confidence interval (CI) using the formula given in the previous sub-section.
- Interprete the OR, and perform the goodness of fit measure of the logistic regression model.

## Results and discussion

The analysis begins by presenting the descriptive statistic of the respondents which covers also information about the prevalence of FCM. Furthermore, we applied binary logistic regression to investigate the factors influencing FCM. This analysis explains factors related to FCM.

### Prevalence of child marriage and descriptive statistics

The descriptive statistics are presented in Table 2. From the dataset, we see that out of 9,333 females, 8,164 (87.47%) were not married before 20 years of age and 1,169 (12.53%) experienced FCM. Although the prevalence is lower compared to the 2012 DHS survey results which was at the rate of 17%, the percentage (12.53%) shows that the prevalence of FCM in Indonesia is still high, where it is targeted to be zero in 2030. Most of the females who participated in the survey had a higher education level (79.74%) and only few of them (0.38%) had no education. Overall, 68.16% were non-working females. The proportion of wealth categories were only slightly different. Most of the respondents lived in West Java (58.78%) and only 9.53% lived in East Java. The proportion of young females living in urban areas was higher than in rural, and most of them had no health insurance (62.95%). The household heads were mostly dominated by males.

**Table 2:** Respondent characteristics.

Variable	Category	Percentage (%)
Current marital status	never in union ever married/living together	87.47 12.53
Highest education level	no education primary secondary higher	0.38 5.85 79.74 14.03
Respondent’s occupation	not working working	68.16 31.84
Wealth index	poorest poorer middle richer richest	23.42 19.79 18.86 18.33 19.60
Province	East of Indonesia Middle of Indonesia West of Indonesia	9.53 33.70 56.78
Type of residence	urban rural	55.04 44.96
Health insurance	no yes	62.95 37.05
Sex of household head	male female	85.42 14.58

### Factors related to child marriage

The results of the binary logistic regression are presented in Table 3. From the table, the logistic regression indicates that education level, occupation, wealth index, province, and type of residence were the factors that significantly influenced FCM (p-values less than 5%). Meanwhile, health insurance and sex of household head did not influence FCM significantly (p-values greater than 5%).

**Table 3:** Results of binary logistic regression model.

Variable	Category	$\hat{\beta }$	SE	Wald	df	p-value	OR	95% CI of OR	
								lower	upper
education level	higher education	0							
	no education	4.337	.482	80.842	1	.000	76.448	29.733	196.707
	primary	4.598	.225	419.080	1	.000	99.300	63.879	154.315
	secondary	2.791	.196	201.958	1	.000	16.296	11.099	23.931
occupation	working								
	not working	.508	.081	39.681	1	.000	1.662	1.418	1.948
wealth Index	richest	0							
	poorest	1.168	.163	51.572	1	.000	3.215	2.336	4.426
	poorer	1.098	.159	47.410	1	.000	2.997	2.195	4.094
	middle	.892	.160	30.984	1	.000	2.440	1.783	3.339
	rich	.663	.165	16.218	1	.000	1.940	1.404	2.681
province	west	0							
	east	.373	.127	8.667	1	.003	1.451	1.132	1.862
	middle	.234	.083	7.965	1	.005	1.263	1.074	1.487
type of residence	rural	0							
	urban	-.332	.083	16.108	1	.000	.718	.609	.844
health insurance	yes	0							
	no	.091	.076	1.425	1	.233	1.096	.944	1.271
sex of household head	female	0							
	male	.172	.106	2.607	1	.106	1.187	.965	1.462
intercept		-21.091	.620	1158.465	1	.000	.000		

The table also presents information about the odds ratio (OR), indicating the association measure of the predictor variables’ category in accordance with the response variable. A value of OR greater than one indicated that the category had a higher prevalence of experiencing FCM compared to the reference category. For the education level variable, the OR value showed that females without education were 76 times more likely to experience FCM than females with a higher education level [OR = 76.448; (CI = 29.73–196.70)]. Furthermore, females with primary education or secondary education tended to also experience FCM more than females with higher education, with the OR = 99.300 (CI = 63.87–154.31) and OR = 16.296 (CI = 11.09–23.93), respectively. Working status significantly influenced FCM, where the probability of non-working females being in FCM was 1.6 times higher than working females [OR = 1.662; (CI = 1.41–1.94)]. The analysis showed that the poorer the woman, the prevalence of being in FCM was greater, indicated by the coefficients (as well as the OR) of the categories which increased with the level of the wealth index. The relative risks are 3.215, 2.997, 2.440 and 1.940 for poorest, poorer, middle and rich family respectively, compared to the richest family. It is interesting to note that the prevalence of FCM for females living in east Indonesia or middle Indonesia was higher than females living in west Indonesia with relative risk of 1.451 or 1.263 times higher for east and middle, respectively. This phenomenon may relate to the economic conditions (growth) of the provinces, where the eastern part of Indonesia is dominated by less developed provinces such as Papua, Nusa Tenggara Timur, etc. Meanwhile, provinces in the west are mostly dominated by large cities such as Jakarta, Bandung, Aceh, etc. In line with this, the negative regression coefficient of urban indicated that females living in rural areas had 1.71 times greater likelihood of experiencing FCM than females living in urban areas [OR = 0.718; (CI = 0.609–0.844)].

The model above resulted in the value of the -2-log likelihood equaling 4979.826, which measured the goodness of the model.

## Discussion

FCM is an important issue that has attracted the attention of the global community due to its consequences, especially in relation to maternal health. Moreover, FCM is also on the SDGs’ agenda, where FCM is targeted to end by 2030. Currently, Indonesia has been listed among the top seven countries with the highest prevalence of FCM in the world, and hence, a study investigating the factors influencing FCM in Indonesia needs to be conducted to support policymaking.

Our findings show a high prevalence of FCM. We found that 12.35% of the young females who participated in the survey were married before 20 years of age. Unfortunately, the raw data did not cover information about the specific age they got married. It may be the case that, among those 12.35% of females, some married before 15 years of age. Our study revealed that there is a strong relationship between the type of residence and the likelihood of FCM. The study also suggests that the likelihood of experiencing FCM for women living in rural areas is higher than those in urban areas. This finding is consistent with the study by Merlo et al. [20] and Ebrahimi et al. [22] indicating that rural inhabitants had a higher likelihood of being in FCM than their urban counterparts. The fact that urban people, as opposed to rural ones, may be exposed to female empowerment initiatives and higher education may be one explanation for this finding. As a result, their attitudes and motivations toward educating female children rather than marrying them off may be altered. Moreover, it can also relate to access to information. Young females (and the family) living in urban areas have better access to information about the dangers and risks of FCM than those living in rural areas.

In contrast to those with no education, our study found that having a secondary or higher education was related to a lower risk of experiencing FCM. This result is in line with several findings from previous studies such as Marshan et al. [17] for Indonesian cases and for other countries see Bolarinwa et al. [5] as well as [21–26], among others. Most studies confirm that education is a preventative measure for FCM. This suggests that revitalizing and enhancing girls’ formal education is one of the best approaches to address FCM in the respective country. Any form of formal education has a large chance of lowering the probability of FCM. Furthermore, if the education of girls is given top priority in Indonesia, that nation will be coming closer to achieving the SDGs and making considerable progress against FCM.

Our findings suggest that young females in a better wealth index have a lower chance of FCM than young females in a poorer wealth index. This outcome is consistent with other research, such as that by Pankhurst et al. [22], who found that poverty is a key factor in FCM because it increases the likelihood of child marriage. Rumble et al. [27] came to a similar conclusion, showing that the likelihood of child marriage was inversely related to affluence. In their study, for the Indonesian case, parents of girls within a poorer wealth index marry off their female children to reduce the financial burden associated with the upbringing of the child. Therefore, parents tend to take any chance of marriage for their female child. This strengthens the postulation that poverty is a critical driver of FCM.

Our study indicates that young females residing in east Indonesia have a significantly higher likelihood of experiencing FCM compared to young females in west Indonesia. In fact, the west of Indonesia is dominated by large cities such as Jakarta, Bandung, etc. The education quality is also much better than in eastern Indonesia. Many young people in Indonesia migrate to Jakarta for work, and most of them have decided not to get married in their youth or even not marry due to career prospects. This also relates to the finding that working females tend not to experience FCM compared to non-working females. Working females may think that they can live independently and not depend on parents or being in a couple. Moreover, many working females have ambitions to achieve a better career path, so that marriage is not a priority [28,29]

The results of this study have important policy and practical implications. In terms of policy, the findings that demonstrate the strong correlation between education and FCM highlight the urgent need for Indonesia to reevaluate its educational policies and places a greater emphasis on mandatory education for girls at all levels. Girls should be given the knowledge about the risk of FCM and hence, the ability to reject FCM. The second priority should be given to poverty alleviation, which is also associated with unemployment. To deal with this, to considerably lower unemployment and poverty in Indonesia, the government and non-governmental organizations (NGOs) must cooperate. By doing this, parents would be able to support their families without having to rely on their daughters getting married and bringing home any cash. The fact that the likelihood of FCM in eastern Indonesia is higher than in the western part is in line with the fact that the government needs to improve the education quality in eastern Indonesia and provide access to better information, especially about the dangers and risks of FCM.

## Conclusion

This study sought to assess the prevalence of and factors associated FCM in Indonesia through a binary logistic regression model. This study revealed that the prevalence of FCM is about 12.59% which is still considered as high. It is critical for policymakers in Indonesia to be aware of the variables that contribute to FCM to successfully diminish or eradicate it, thus supporting the SDGs’ target. To achieve the SDG’s target, serious interventions need to be formulated which directly targeted the factors which significantly increase the likelihood of FCM. This study identified that education and poverty alleviation through job allocation for girls were some protective variables that may be reinforced. The level of education was found to be the most significant variable as a driver of FCM in Indonesia. Designing specific education programs (either formal or informal education) could be one of the strategies to decrease the FCM prevalence. Moreover, living in the east of Indonesia and a low level of education were some other controllable factors that increased the likelihood of FCM. To deal with this, community development programs or the interventions programs need to be focused for families living in the eastern part of Indonesia. Moreover, the government should allocate more budgets for education improvement in eastern part of Indonesia such as by designing targeted scholarship program. Building networks with Non-Government Organizations (NGO) focused on women could be an effective way to speed up the alleviation program.
